# Disaster impacts, resilience, and sustainability opportunities for Gili Trawangan, Indonesia: transdisciplinary reflections following COVID‐19

**DOI:** 10.1111/disa.12554

**Published:** 2022-07-04

**Authors:** Stefan Partelow, Marie Fujitani, Sian Williams, Delphine Robbe, Raditya Andrean Saputra

**Affiliations:** ^1^ Leibniz Centre for Tropical Marine Research (ZMT) Bremen Germany; ^2^ University of Bremen Germany; ^3^ Gili EcoTrust Gili Trawangan Indonesia; ^4^ Indonesia Biru Foundation Lombok Indonesia

**Keywords:** capital, collective action, community, COVID‐19, earthquake, island, social capital, Southeast Asia, tourism, transformation

## Abstract

This article provides transdisciplinary reflections from scientists and local NGO managers on the international small‐island tourism destination of Gili Trawangan, Indonesia, concerning the impacts, short and long‐term adaptation strategies, and sustainability opportunities from two disasters occurring in rapid succession: the 2018 Lombok earthquakes and the COVID‐19 pandemic starting in 2020. A brief review of the island's governance challenges sets up our analysis of revisited findings and new empirical insights on how the island's communities have dealt with two unique disaster scenarios over the last three years. We draw on a community resilience framework premised on social capital and collective action theories to position the island's ability to transition towards sustainable tourism post‐pandemic. We conclude with sustainability opportunities going forward.

## 1.0 Introduction

Since the early 1990s, Gili Trawangan, a small island off the coast of Lombok, Indonesia, has grown from a few pop-up bungalows to a world renowned tourism destination focused on SCUBA diving local coral reefs. Before COVID-19, the island was receiving up to 1 million tourists per year, supporting thousands of local livelihoods and hundreds of businesses with more than 30 SCUBA centers. Historically, the island has faced numerous disasters including volcanic eruptions and earthquakes (Partelow, 2020). Recently, a series of large earthquakes in August 2018 and the on-going COVID-19 pandemic have brought extensive challenges to the tourism economy and viability since the end of 2018. While there have been many negative impacts, there are also opportunities to rebuild and rethink the island's trajectory and governance strategies.

In this article, community resilience (Faulkner et al., 2018) is examined in the context of recent disaster impacts leading to governance challenges and opportunities for sustainable development of the tourism sector in the aftermath of COVID-19. Globally, tourism development on small-islands, particular those in the tropics, face unique challenges due to the likelihood of isolation from mainland infrastructure and society (Peterson, 2020; Zhang and Managi, 2020; Walker et al., 2021). Collective action and community-centered development initiatives are often critically important processes for addressing basic problems and challenges, as well as ensuring that tourism initiatives can meet livelihood needs while maintaining the cultural identity and environmental integrity that often attracts tourists and ensures local wellbeing. As a result, scientific interest includes a wide variety of topics on sustainable tourism, with current efforts often employing inter- and trans-disciplinary approaches that work together with communities and local development initiatives to better understand and catalyze sustainability transformations (Hind et al., 2015; Glaser et al., 2018).

This article has been accepted for publication and undergone full peer review but has not been through the copyediting, typesetting, pagination and proofreading process, which may lead to differences between this version and the Version of Record. Please cite this article as doi: 10.1111/disa.12554.

This article is protected by copyright. All rights reserved.

More specifically, community resilience is needed for effective disaster response and recovery in the absence of external aid from states, non-profit organizations or private donations (Paton, 2003; Nakagawa and Shaw, 2004; Brown and Westaway, 2011; Berkes and Ross, 2013). Even when external aid is available for disaster relief, it cannot directly provide the social capital, emotive and cognitive foundation needed to rebuild well-being from within (Aldrich and Meyer, 2015; Sadri et al., 2017). Analyzing community resilience positions internal capacities as the core features enabling short and long-term recovery towards sustainable development (Berkes and Ross, 2013; Faulkner et al., 2018; Partelow, 2020). By spotlighting Gili Trawangan, we intend to provide an accessible synthesis of existing research and practical experiences, along with an example that broadly reflects many other small-island tourism destinations in Indonesia and in emerging tropical economies around the world.

This article provides a synthesis of the main impacts and opportunities from recent disasters on the island, and an overview of the factors contributing to community resilience (Faulkner et al., 2018). Our insights are derived from a review of the literature, empirical findings and transdisciplinary collaboration with two local non-governmental and non-profit organizations working locally, the Gili EcoTrust (http://giliecotrust.com/) and the Indonesia Biru Foundation (https://indonesia-biru.com/). Our results are presented in two sections. First, in section 3.1 we descriptively detail the disaster impacts and sustainability opportunities by topic and over the time frame of both disasters since 2018. We then present our analysis of the five features of community resilience in section 3.2, which include (1) place attachment, (2) leadership, (3) community networks, (4) community cohesion and efficacy and (5) knowledge and learning.

### 1.1 Tourism development on Gili Trawangan, Indonesia

Effective place-based governance is a well-understood necessity for managing shared resources and provisioning public goods in a sustainable and equitable manner (Davidson and Frickel, 2004; Lemos and Agrawal, 2006; Partelow et al., 2020). Gili Trawangan is no exception. Some of the management and governance challenges on the island include waste collection, processing and recycling, infrastructure building and maintenance (water, electricity, docks, moorings, roads), coral reef and environmental health and habitat restoration, as well as public health and safety for tourists and residents. Historically, governance of these resources emerged through collective action among private tourism businesses and local governments (heads of island and regional authorities) (Graci, 2013; Partelow and Nelson, 2018).

Since 2002, a local NGO called the Gili EcoTrust has taken a key role in facilitating self-organized governance by local businesses through building trust, community networks and institutions for collective action among the island's stakeholders. The organization has developed and maintained rules, norms and action-oriented projects linked to conservation and sustainability activities, including: community beach clean-ups, coral restoration projects, nesting sea turtle protection and awareness, ecotourism information sharing, animal welfare campaigns, and tree planting along with waste collection and recycling services led by another local Indonesian organization called FMPL (Front Masyarakat Peduli Lingkungan - Community Front for Environmental Care) coupled with advocacy and

This article is protected by copyright. All rights reserved.

Accepted Article awareness campaigns for recycling and reusable product consumption. The Eco Trust is funded through public donations, project grants and voluntary contributions from participating SCUBA businesses who give diving customers the opportunity to donate a one-time amount (~USD 1.50 USD) that directly supports Eco Trust activities.

National government public services have funded larger infrastructure projects such as a recent solid waste processing facility, roads, local police, a pier and sewage systems. The island has a mandate against motorized vehicles, with all transportation done by single horse pulled carts (Cidomos). Only when larger government projects are conducted, motorized vehicles are brought on to the island temporarily if needed. Local government (regency level) is tasked with managing day-to-day operation of the newly constructed waste facility, as of October 2020 they have sent two people which is currently insufficient for the workload. Similarly, the sewage system is built but not yet operational or managed by the local government. Taking these examples as a broader trend, tensions often exist between the self-organized efforts of local businesses along with Eco Trust and the regional government. Governments have finances and authority to make decisions but may be disconnected from understanding what solutions will work locally and may lack incentives to act in a timely manner, while local stakeholders may know what needs to be done but often lack the financial backing or authority to move forward on urgently needed projects. Local families involved in island politics have historically had substantial influence over island governance and island-government relations often linked to the success of their tourism businesses and stakeholder connections. Governance cooperation between all involved in a way that builds trust and transparency has been difficult, especially when many of the resident business owners (de facto; legally only business partners) are foreign citizens who bring in substantial tax revenue and employment, but receive little public investment in return or knowledge of where taxed revenue is allocated in government spending (Partelow and Nelson, 2018) or whose practices could lead to disproportionate economic leakage from tourism (Smith and Jenner, 1992).

A review of peer-reviewed literature examining tourism on Gili Trawangan indicates that social capital and informal community networks leading to self-organized collective action and business-NGO-government partnerships have been important factors shaping marine space and waste governance, economic development (permits, taxation, infrastructure) and sustainability outlooks (Kamsma and Bras, 2000; Satria et al., 2006; Graci, 2008; Bottema and Bush, 2012; Charlie et al., 2012; Graci, 2013; Rianto, 2014; Hampton and Jeyacheya, 2015; Partelow and Nelson, 2018; Partelow, 2020). Waste governance has been a continual challenge regarding how collection and processing should be organized, who should do it, and where the waste should go (Dodds et al., 2010; Willmott and Graci, 2012; Nelson et al., 2019), which has shifted responsibilities and funding sources over the last decade. The ability to change behavior to be more environmentally friendly through informational nudging has been examined on reducing single-use plastic and decreasing reef impacts (Nelson et al., 2021a), paying for marine conservation (Dodds et al., 2010; Nelson et al., 2019) and paying for green hotel certifications (Nelson et al., 2021b). Network research has shown the island's social and ecological interconnectivity including patterns of reef use (Eider et al., *under review*) and business collaborations (Partelow and Nelson, 2018). The island is also undergoing landscape changes, coastal erosion, increased infrastructure development and coral cover changes (Kurniawan et al., 2016a, b). The island's impacts and cooperative efforts to

This article is protected by copyright. All rights reserved.

Accepted Article recover following the August 2018 earthquakes indicates a high level of community resilience following a short-term local disaster (Partelow, 2020). Numerous additional articles about the island have been published on cultural and economic impacts (Diah Sastri Pitanatri, 2018, 2019), critiquing ecotourism (Halim, 2017), breakwater protection (Pradjoko et al., 2015) and religiosity (Varga et al., 2018).

**2.0 Methods**

The findings in this article synthesize existing literature related to Gili Trawangan and new empirical research from the academic authors (SP, MF) co-produced with transdisciplinary partners in practice (SW, DR, RAS).

Peer-reviewed literature was collected from SCOPUS and Google Scholar searches on search terms linked to the island (Gili Trawangan, Gili Matra, Gili Matra Marine Park, Gili Air, Gili Meno, Gili Islands). All articles found were scoped to exclude unrelated articles, and then read (N=35) and inductively coded with the main emergent themes on disaster and sustainability issues (Table 1). When relevant, content was coded into the Faulkner et al., (2018) frameworks of five factors for community resilience: (1) place attachment, (2) leadership, (3) community networks, (4) community cohesion and efficacy and (5) knowledge and learning (Table 2).

The article also draws on insights from over 100 semi-structured interviews conducted by SP in prior research on Gili Trawangan on topics of sustainable tourism and resilience, in part regarding the aftermath of the 2018 Lombok earthquakes (Partelow and Nelson, 2018; Nelson et al., 2019; Partelow, 2020). In addition, 11 key informant interviews were conducted from October - December 2020 with local business owners and community leaders who remained on the island despite the continuing economic hardship from lack of tourism and ban on international travelers entering the country (SP, MF). These interviews included follow-up questions and reflections on how individuals, the tourism community, and formal and informal governance structures responded and adapted to the two different successive disasters and the current impacts of COVID-19 on the community. Interviews were conducted over the phone due to travel restrictions. Interviewees gave prior informed consent, and all interviews were recorded, transcribed and coded for emerging themes on COVID-19 impacts, challenges (and strengths) related to economic, environmental, and social sustainability, and governance related issues (Table 1). The qualitative data analysis software MaxQDA (20.2.2) was used to organize the coding process. Nonetheless, conducting interviews remotely comes with its own set of challenges including scheduling, time changes and connection difficulties between Indonesia and Germany, as well as the inability to read facial expressions and body language which can impact the interpretation of statements on both sides. Trust is also needed to speak, often with people you have never met in person, about personal topics related to hardship and loss. However, there are also advantages such as high quality recording, advanced scheduling and flexibility over a period of time that may be longer than in-person field work travel.

Insights in this article are also derived from transdisciplinary partners and co-authors, the Gili EcoTrust (http://giliecotrust.com/) and the Indonesia Biru Foundation (https://indonesia-biru.com/). Transdisciplinary research refers to co-produced research between academic and non-academic

This article is protected by copyright. All rights reserved.

Accepted Article partners, and is increasingly recognized as important approach for generating locally relevant and practically applicable findings (Max-Neef, 2005; Lang et al., 2012; Thompson Klein, 2014; Polk, 2015). The partners in both organizations have decades of experience living and working on the island and in the region addressing sustainable development challenges as practitioners. The on-going partnerships include multiple ongoing projects, including this synthesis of disaster and COVID-19 impacts. Synthesized insights in this article were co-generated and co-written as part of a joint activity. The purpose of the current activity is to generate place-based insights from science and local knowledge that are locally meaningful and grounded in practice. Nonetheless, as authors, our positionality plays a role in our engagement in the case study area, understanding of the challenges and opportunities, and ability to make any authoritative statements about the island, its history, diversity of people and potential trajectory. The academic authors (SP, MF) are citizens of the United States, and are employed at German higher education institutions. The authors (SW, DR) have European backgrounds, and have lived on Gili Trawangan working on direct conservation and development initiatives for well over a decade. (RAS) is an Indonesian national, living and working on Lombok on marine conservation issues with several years of experience in marine conservation on the Gili islands. A core purpose of such research partnerships is to link different perspectives. Though no position is bias-free, being close to a topic always risks potential biases due to personal attachment that needs to be reflected on as knowledge is co-produced. These issues are well-known in transdisciplinary sustainability science, where there is a growing literature on roles and processes of self-reflection on researcher and practitioner positionality, as well as the positive aspects of internal and local agenda-setting (Wittmayer and Schäpke, 2014; Horlings et al., 2020; Breckwoldt et al., 2021).

## 3.0 Results

### 3.1 Disaster impacts and sustainability opportunities

Five major themes emerge as important when synthesizing disaster impacts and sustainability opportunities on Gili Trawangan following the August 2018 earthquakes and COVID-19 (Table 1). First, are the responses and adaptive strategies of the local Indonesians, who are perhaps the least studied, academically recognized and understood social group on the island. While there are an estimated 200 local Indonesian households on the island, more than 2000 local Indonesian employees commute from Lombok to work in the service sector. Informally, the impact of the Gili Islands on the north Lombok economy is perhaps being the primary employer beyond fishing and farming. The August 2018 earthquakes shattered this income opportunity, as tourism was shut down and most returned to Lombok for family and community grieving and rebuilding activities. However, COVID-19 mobility restrictions have posed a larger long-term challenge for tourism livelihoods. Many businesses, as during the earthquake closures, continued supporting employees on payroll, but this couldn't be done indefinitely during the pandemic. Many locals who were primarily employed in tourism reportedly shifted to fishing or farming on family land as an adaptive livelihood strategy. This strategy, in contrast, was not an option for foreign employees and business owners.

The second theme involves the responses and adaptive strategies taken by people working in the tourism sector with a focus on those day to day operating businesses. The tourism sector is largely de-

This article is protected by copyright. All rights reserved.

Accepted Article facto owned and managed by Western nationals, many who have been living and operating on Gili Trawangan for more than a decade, although there had been an increasing number of east Asian businesses and Indonesian investors (non-local) up until COVID-19. Small hotels and retail services continue to require foreigners to be in partnership with cooperatives or Indonesian Micro, Small, and Medium Enterprises (Usaha Mikro, Kecil, dan Menengah or "UMKM"), while as of 4 March 2021 full foreign ownership of high-end resorts and some categories of large businesses has become possible (Presidential Regulation No. 10 of 2021 on Investment Business Sectors). However, these legal developments are too recent to be relevant to the time frame of the current study. The requirement for foreign partners to run businesses only in cooperation with Indonesian partners who retain formal ownership applied to the businesses during this study, and may have contributed to the sense of control and belonging experienced by foreign business de-facto owners through the disasters. The earthquakes left many businesses needing repairs, but the physical damage was mostly short term although many individuals incurred psychological trauma. Nonetheless, 2019 saw a near return to full tourism after successful self-organized efforts by remaining locals and business owners to rebuild, clean and prepare the island for reopening 2-3 months later. COVID-19 has been different, the social capital and local self-organizational capacity was less helpful in buffering the island to international travel shutdowns, which closed 90% or more of the businesses. As of June 2021, after 14 months, tourism on the island remains at peak lows with only domestic tourists occasionally visiting the few open services.

Third, environmental impacts with focus on coral reefs and near-shore health, remained resilient to earthquake impacts with a few incurred cracks and damage from physical shaking, but reported and anecdotal increases in marine life attributed to the absence of heavy boat and SCUBA traffic accruing otherwise all day year round. During the pandemic, the more than yearlong removal of tourists and boats has brought increased observations of marine megafauna on and around the island including nesting sea turtles. Informal reports of more abundant marine life have been reported along with increased reports of fishing and egg poaching by unemployed locals, however. Well established coral restoration projects continue to run on the island’s eastern shore. Although short term impacts on the island’s reef may be getting a much needed break from human pressure, long term concerns include increased sea surface temperature driving coral bleaching which the local reefs have faced in 2016 and 2017.

Public health following the 2018 earthquakes through COVID-19 has remained a perennial concern. As a small island, few supplies and clinics are directly available on the island. During the earthquakes, immediate first responder aid was pieced together by residents with prior medical and first-aid training, needing to evacuate serious injuries off the island. During the pandemic the island remained case-free for most of 2020, although reports of cases in 2021 have emerged. Although few cases have been reported, partly to enable reopening as soon as possible, testing frequency and treatment centers in the one clinic that has remained open raises public health concerns, particularly for the most vulnerable local households with less ability to access needed resources compared to foreign nationals, for example, in Bali or Mataram. In addition, few standard operating procedures exist to deal with disasters such as earthquakes, beyond learned experience. Local schools have worked with children to overcome trauma, but few services for others exist.

This article is protected by copyright. All rights reserved.

Accepted Article

Finally, community cohesion and social capital are an essential component of the island's functionality due to the self-organized nature of governance and problem solving that arose during its early development until today without substantial government involvement. Nonetheless, growing over the last decade, this has fractured into subgroups under a now different group of foreign nationals than in the 1990s, often with different views, personal motives and investment strategies (personally and financially). During the earthquakes, local Indonesians almost exclusively fled to Lombok. Many foreigners also left to either Bali or Lombok (to provide support from there in supply acquisition and fundraising) or back to their country of origin permanently. Joint collective action among those who stayed on the island or in the region created social capital, importantly, between local Indonesians and foreign nationals. This further fostered belief in the ability of the community to work together and resolve its own problems, although these events were not without distress and disagreement. Working together during the pandemic is different. Dire outlooks coupled with lack of a sense of control or foreseeable end, left many businesses closed (a likely high but unknown number permanently).

**Table 1**. Synthesis of disaster impacts and sustainability opportunities on Gili Trawangan following the August 2018 earthquakes and COVID-19. Overall, each presents challenges for cooperative governance. Each focal area is an action arena for institutional development and change to address sustainability goals and challenges.

| **Focal areas** | **Impacts from 2018 earthquake** | **Lessons learned from earthquake** | **Impacts during/ from COVID-19** | **Opportunities for sustainability** |
|---|---|---|---|---|
| **Timeline** | **August 2018** | **2019** | **2020-2021** | **Future** |
| Local Indonesian livelihoods | ~2000 local jobs impacted from Lombok for 1-3 months, some never returned. Many working with businesses to rebuild and prepare tourism return. | Tourism is still a viable employment sector. Aid received from Indonesia Central Government and international orgs, allowing quick tourism recovery. | Difficult for many ~200 local households impacted, more fishing and farming. ~2000 Lombok commuter jobs impacted, more work in farms/small shops. | Income diversification needed. Less short term tourism work, more long term. Local governments start job programs on sustainable fisheries, monitoring, and conservation. |
| SCUBA & tourism businesses | Tourism closed for 1-2 months. Rebuilding for many. Local employees left. Excavators and trucks rented. Many foreigners left permanently, some businesses too. Collective action by many for revival. | Dive operators and the local community can work together to promote tourism safety. Experience gained, and social capital built for the future. Community networks are stronger. | 90% of businesses closed for >1 year. Foreigners (~2000 pre-COVID) on the island reduced ~85% day-to-day. International tourism was blocked, domestic tourism remained very low. | Fewer businesses will lower competition, increase water safety, decrease transaction costs for communication. Fewer businesses will reduce environmental impact on the island, making cooperation easier. |
| Environment & coral reefs | Cracks and landslides in several | The reefs are resilient. There is no | More fishing by some locals. More | Reduced SCUBA pressure on reefs |

This article is protected by copyright. All rights reserved.

Accepted Article

| | | | | |
|---|---|---|---|---|
| | dive sites. Increased reef life presence. Short-term reduction in noise pollution from boats and diver impacts. Less tourism waste but disaster rubble. | significant change in coral cover and reef fish abundance. | biodiversity on some reefs with less boats. More turtle landings and nestings. Increased turtle egg poaching by unemployed locals. | (presence, noise) Less waste being produced, including wastewater management. Regular beach clean-ups. More awareness efforts. Fewer businesses will lower pollution. Established waste collection system. |
| Public health & education | Both locals and expats developed trauma. Some severe, leaving the country. The primary school on Gili Air closed for > 3 months. Teachers and parents did not want kids under unstable roofs. Piles of rotten food from hotels and restaurants, more rats and disease risk. | No Standard Operating Procedure for pre, during, or post disaster. No mitigation plans before, e.g., safe building rules. Teachers spent 2-3 months mitigating trauma with local children. School mitigation protocols. | All small unofficial medical clinics and pharmacies closed. Main legitimate clinic closed for +1 year. One clinic opened for the island with a part time registered doctor, one with corona testing. Many fear catching covid from hospitals or doctors. | More buildings with earthquake-friendly design, adopting local styles with wood, bamboo and light roofs. Government funds for sustainable buildings. Info campaigns for waste reduction, nightlife safety, animal welfare. Protocols for disaster response. |
| Community cohesion & social capital | Most locals first fled to Lombok, leaving local businesses and the island with few. Remaining survivors self-organized supplies, rebuilding, coordinating future plans. People are living day by day. | Tourism came back, only ~3 months after. Global tourism demand remained high despite aftershocks for months. Trust gained in working together. Place attachment high, aiding collective action. | Many foreign owners/ employees left, some permanently. Limited domestic tourism hurt the dive industry. Some local warungs spared with Lombok tours, but most closed. Living week by week on an empty island. | Less crowded and smaller communities to rebuild the economy and resolve governance issues. Higher bonding and bridging social capital, less conflict. Long term investors with place attachment. |

This article is protected by copyright. All rights reserved.

Accepted Article

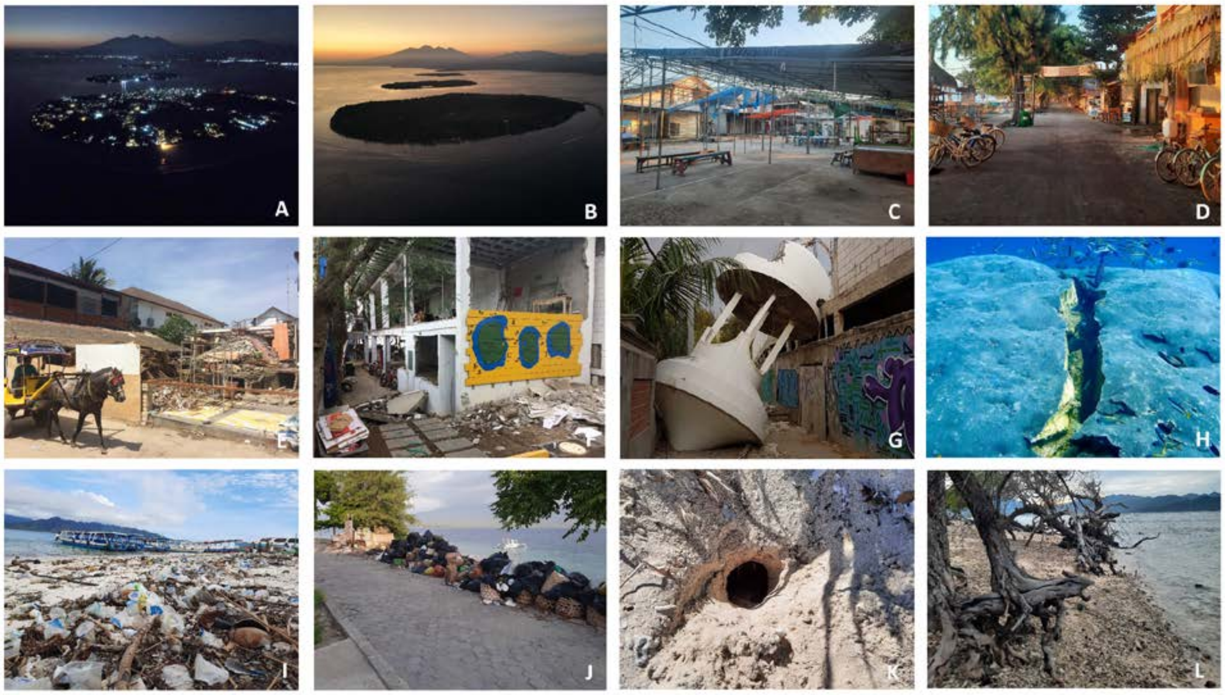

**Figure 1.** (A) Sunrise of lighted Gili Islands, north Lombok coast and Mount Rinjani pre-pandemic. (B) Sunrise of dark Gili Islands, north Lombok coast and Mount Rinjani during the pandemic shutdowns. (C) Empty night food and night market during pandemic, a central gathering place. (D) Empty main street on east side of Gili Trawangan. (E) Earthquake rubble and horse cart following 2018 earthquakes. (F) Damaged building post-earthquake 2018. (G) Toppled mosque spire post-earthquake. (H) Cracked coral head off Gili Trawagan post-earthquake. (I) Plastic litter covering the beach. (J) Waste piles gathering on shore. (K) Signs of turtle egg poaching during the pandemic. (L) Coastal erosion, a continual problem. All photos from authors.

### 3.2 Beyond COVID-19: Sustainability opportunities through community resilience

Community resilience on Gili Trawangan is an interactive and fluctuating entity, drawing on prior experience among locals and foreign nationals to provide leadership and adaptive strategies to deal with new problems. Translating the relatively high level of community resilience built through the 2018 earthquakes into dealing with the pandemic has been challenging. Furthermore, speculating how the island will adapt going forward to address new and recurring sustainability challenges is unknown. Nonetheless, the adaptive learning on the island over the last decade provides an opportunity to merge empirical findings and practical experiences with a theoretical lens of community resilience. This allows insights to be made about how the island's context matches with more general features that enhance or decrease resilience from the literature, to transfer and reflect on lessons learned. We situate our above synthetic findings into the five capacities of community resilience identified by Faulkner et al., (2018) which include: place attachment, leadership, community networks, community cohesion and efficacy, and knowledge and learning (Table 2). Direct quotes from interviews are provided below the synthesis of each capacity.

This article is protected by copyright. All rights reserved.

Accepted Article

1) **Place attachment**: A strong driver of collective action for those who stayed after the earthquake to rebuild faster and with deeper social connections. Similarly, in the early months of the pandemic many businesses stayed and supported staff at a financial loss. Many feel a strong connection to the island and a commitment to see things through. Place attachment broke for some who took it as an opportunity to leave the island for something new, and for others who could no longer afford to stay. Long-term unknowns are simply economically demanding and require external forces beyond self-organized collective action to solve. Though place attachment can create over-optimistic attitudes that undermine acceptance of harsh realities or practical strategies going forward, conversely it is a motivating factor for adaptation. Those who stayed have a deep attachment, financial, personal and social investments, hoping the island can recover into a better version of its tourism economy with longer stay, place-based tourism.

"*some businesses that grow big…they have managers running their businesses here, those are normally the ones that could easily sack everyone…because they are not emotionally [involved]…they're not there, the ones that are sticking through*"
   - Indonesian business owner

*"the expat community only really started leaving after like, a couple of months…there's no support networks… but it's kind of kept this community vibe going. But at the time, they were thinking that they were just going home and forget their money, have a little break…and just come back in two months' time… when people really started to leave then then it got strange, and now loads of people have left."*
   - Local NGO manager 1

2) **Leadership**: Local within-community leadership was critical during the earthquakes, as many individuals rose to the occasion, using personal skills, financial and informal social capital to foster collective action among residents. Cleaning, repairing, fundraising, outreach and hosting isolated residents were tasks willfully taken on by many and embraced by nearly all who could help. The pandemic requires a different form of leadership. Consolidation of the economy, participation in decision-making and the politics of reopening, advocating for the use of public funds to rebuild and regenerate tourism requires an engagement into the region's tourism political economy between government and community stakeholders that has historically faced communication and transparency challenges. The recent dual disasters have pulled into focus who in the community is dependable in terms of support and mobilizing action in a crisis. The identification of strong leaders is an important predictor of collective action and resilience moving forward.

*"They are the ones that are the most actively engaged…I would call them the hardcore ones that are still here…there's always a handful of us, the main five or six shops that have been here the longest. I really agree with them and we call it mini GIDA, basically you know if something comes up and you've been asked to deal with it, and you're not really sure how to do it, as the head of*

This article is protected by copyright. All rights reserved.

Accepted Article

*GIDA, you could sit down with these six people, it's not really Mafioso style, its more to support you than the other way around, but yeah they are the elders."*

- Foreign SCUBA business owner 1

*"A lot of people panic, in any situation, which a lot of people have done, you know, some financially, some emotionally some, because they can't handle it…and we've learned a lot from the earthquake, then a lot of looking after each other watching each other's backs [within the core community]…I suppose we've just been trying to spread the wealth of that…. But we've spoken quite a lot, a lot of the big boys on the island, I think it could be an amazing reset for the island…There was too many people here, people have just paid the right people to be able to come in and do whatever the hell they want, build whatever the hell they want. Those sort of people, and it just can be a wonderful reset."*

- *Foreign SCUBA business owner 2*

3) **Community networks**: Earthquake recovery made many community networks stronger, and some disagreements among subgroups more clear. Nonetheless it was overall positive. Community networks, formal and informal, have helped many overcome the social-psychological difficulties of lost livelihoods and vibrant social connections that characterized daily life on a small tourism island before. Those who stick through pandemic reopening, will likely emerge with strong networks building on both disaster experiences going forward.

*"the community you get to know a lot more people…so that's a good side of it for everyone here…the main of the community is still definitely here…when you pass them on the street, there's still lots of hellos and smiles and stuff. So that's good…[if] I'm having a bad day, my mate, pulls me out, and then vice versa. It's proving to me that it's gonna take a lot to crack the community."*

– Foreign SCUBA business owner 1

*"My friends are all managers and owners of hotels and restaurants and bars here. So you're all one community together, here [in GIDA], we are the dive centers, all forced to sit with each other so that's why everyone connects with each other one way or another. So I think because of that everyone is kind of connected more."*

– Foreign SCUBA business manager 1

4) **Community cohesion and efficacy**: The immediate post-earthquake community response demonstrated that the community can act quickly, cohesively, and effectively to meet severe challenges. Tourism growth since 2010 and reopening quickly after the earthquakes gave the island's residents and employees a sense of self-control, reaffirming that collective local investments can create a prosperous tourism recovery. Cohesion and efficacy, coupled with previous successes with bottom-up collective action has motivated sentiment that increased empowerment through recognition of decisions from local collective-choice mechanisms could enhance adaptive capacity (Ostrom 1990) as well as increased management autonomy. COVID-

This article is protected by copyright. All rights reserved.

Accepted Article

19 has left many questioning the future of the island, undermining this sentiment, and revealed vulnerability to a wider range of challenges linked to sustainable development. On the other hand, consolidation and scaling down can also be positive for social cohesion, cooperation and environmental impacts if the efficacy of reopening and rebuilding is taken as a community building activity rather than an individualistic one.

*"Yeah, 100%. [Once the pandemic hit] everyone kind of worked. Everyone kind of went okay. No worries. Like, I think everyone just jumped to it. Because I think we're so used [to disasters]. In regards to like learning, like from the earthquake, I think we, I think the island did well to still kind of come together."*

- Foreign SCUBA business manager 2

*"I think people are trying really hard [to stay positive during the pandemic]. There's a lot of community spirit, people take it together. This can be quite a lot of partying…So this part is going on for them. But I'm assuming that's normal. But I think it's also a way for people to come together… I think it's a grateful Island. It's a cool place. And I have only positive things to say about the expat community. I mean, since we've experienced this only being really positive."*

- Foreign tourism business owner

5) **Knowledge and learning**: Learning through both disasters has been continuous, but in different ways. Recovering from the earthquakes was known to be possible, and lessons learned were practical and clear: standard operating procedures, enforced construction codes and place-based building techniques and materials, reefs incurred minor damage, medical supplies, and global support are needed to be resilient to future events. Communication channels were opened between different groups not previously in regular communication (including government, NGO and businesses) through effective use of technology (WhatsApp/Facebook); these channels remained open and were critical to the spread of knowledge during the pandemic. The social memory of the cohesive and collective response to the earthquake influenced actions by members of the community at the onset of the pandemic. However, as the pandemic drags into its second year, learning has turned introspective, long-term and social-psychological: what is the island's future? What will tourism economies be like post-pandemic? How can I create a good future for myself and the island? Local knowledge and experience, and the passing on of that knowledge to new residents and into new situations, has played a continual and important role in the island's past prosperity. It is unclear if post-pandemic development will be a side-step, a step-back or provide a fresh set of insights for how tourism development will continue going forward.

*"but we know we've learned so much in terms of common collaboration and working all together to make the island better during the earthquake. So let's set up those things [again]."*

- Foreign environmental NGO manager

This article is protected by copyright. All rights reserved.

Accepted Article

*"This is a reset button if we can prove that…different concepts of tourism, better tourism, exclusive tours, and [are] better. Yeah. This is a first experiment. Maybe we can have different policies in tourism. Now, we have a chance to actually take data…then maybe we can learn we can restructure tourism."*

- Indonesian conservation NGO manager

**Table 2.** Capacities decreasing or enhancing community tourism resilience on Gili Trawangan. Community resilience capacities adopted from Faulkner et al. (2018).

| **Community resilience capacities** | **Description of capacity** | **Factors decreasing resilience** ↘ | **Factors enhancing resilience** ↗ |
|---|---|---|---|
| Place attachment | Affective, cognitive, and material relationship with place | Conflict among expats and local people, with large economic and cultural gap. Covid crisis is too long and people are losing trust that tourism will resume. Overcrowding, loss of local feel, negative press on pollution and crowds. | Fair business and investment climate. Education level of local people. Working together on decision-making and governance. Cooperation and trust gap smaller since earthquake rebuilding. |
| Leadership | Leaders, organizations, characteristics, roles, and actions that affect outcomes | Lack of government leadership and communication. Lack of enforcement of rules/regulations for safety. Businesses with different motivations. Lack of transparency and trust in decision-making. Elite families and owners having non-transparent political power. Government buildings left without management, locals not allowed to help. | Self-organized leadership is strong. More power to enforce laws. Increased cooperation, communication, and transparency among stakeholders. Strong place attachment by leaders and local knowledge of history and recurring issues. Custom awig-awig (local rules) governance among local families. |
| Community networks | Bonding and bridging ties enabling collective action | Short-sighted individual actions among the communities for profit or market advantage. Past conflicts never resolved. Trust decreases with lack of transparency in decision-making. | Strong informal networks; small island, most know each other and have common goal for thriving tourism, and believe they can be successful. |
| Community cohesion and efficacy | Ability to act together; belief, trust, and empowerment in performing and managing situations | Within group conflicts and disagreements about funding and strategies. Pride and past mistakes blocking discussions and decisions for a better future. Lack of ability to formalize community agreements and enforce removes a sense of self-control in governance. | Disasters fostering cooperative relationships and quick responses by members of the community increases confidence in ability to manage crises (Clinic, excavator, trucks, schools). Fewer people and tourism brought local and foreign communities together with stronger engagement. |
| Knowledge and learning | Individual and group capacity to respond to | Lack of learning facilities (learning centers, teachers, teaching materials). Tourists coming from all cultures and countries, different norms. Lack of | High quality learning centers. Information and outreach by local NGOs. Communication networks for sharing information and |

This article is protected by copyright. All rights reserved.

Accepted Article

| | | | |
|---|---|---|---|
| | local needs and issues through learning and social memory | investment and incentives into cooperative governance and transparency. | transparency (e.g., for disasters, business startup, infrastructure). Government, NGO and business communications higher. Effective use of technology (WhatsApp/facebook). |

**4.0 Discussion and conclusions**

Transdisciplinary approaches have been useful in this analysis for gaining place-based knowledge that is co-produced and contextually embedded for rapid assessments of community disaster resilience. The Gili Trawangan community has embraced disasters as an opportunity, continually reconfiguring its social and governance organization to adapt to new challenges. However, the question of 'who governs the island?', formally and informally, remains contested, politically sensitive and a day-to-day learning and re-learning process among all involved. The Gili EcoTrust has been a consistent self-organized effort throughout the island's many changes and challenges, driving much of the maturation of the community resilience capacities outlined above. Nonetheless, the end of COVID-19 is not yet in sight and international tourism is not returned to pre-pandemic levels (as of April 2022), and the consolidation of the island's businesses and tourism flows have paused many environmental impacts but also threatened all reliant livelihoods. One major question is whether the race to reopen tourism and reboot the economy will catalyze or undermine cooperation efforts among businesses, NGOs and local government on much needed environmental and social welfare issues. This article has shown that the island's current capacities for community resilience are maturing, and at the very least provide a set of enabling conditions built on past experience to contribute to sustainable development going forward.

Community resilience remains an important conceptual framing for examining the success of short and long-term disaster relief and recovery processes. The factors enabling successful preparedness, response and recovery to disasters are increasingly recognized as internal, those derived from and shaped within communities (Adger et al., 2005; Brown and Westaway, 2011; Berkes and Ross, 2013; Aldrich and Meyer, 2015). Nonetheless, external factors also shape community resilience on such a small island such as social media coverage, international donations and support and aid from local and national governments. For example, Gili Trawangan's extensive network of prior foreign travelers provided financial donations and awareness during disaster events that provided much needed aid and motivation for local mobilization. Importantly, disasters can be framed as opportunities and catalysts for sustainability transformations because they can reconfigure approaches to local governance challenges, strengthen community social organization and capacity, or to realign local development towards sustainability goals. On Gili Trawangan, COVID-19 will allow for reconfiguring who is on the island, involved in politics and invested in the island's future. Those who remain throughout the pandemic period, will be part of the island's community who have faced a new challenge and emerged from it with a new sense of resilience going forward. The pandemic also offers rethinking the island's

This article is protected by copyright. All rights reserved.

Accepted Article development trajectory, down-scaling and reorienting business and governance cooperation strategies that can allow a more sustainable and resilient future in the long-term.

Transdisciplinary partnerships can help bridge the gap between academic findings and what is needed in practice for creating change processes that fit local contexts (Lang et al., 2012; Polk, 2015; Lawrence, 2020). This is particularly helpful when formulating insights that can align with local political, cultural, and contextual realities so they can be better taken up. However, it is not without challenges. Engaging in transdisciplinary partnerships positions researchers more firmly into the systems being studied, as subjects of change themselves, which requires ethical and methodological reflection on the positionality of all involved.

Importantly, this analysis only examines Gili Trawangan, one of three neighboring islands, all situated within the broader political economy of tourism in the Lombok and Bali region. The islands are located in the Gili Matra (Meno, Air, Trawangan) Marine Park, a zoned multi-use Marine Protected Area (MPA) designating tourism, conservation and fishing areas. The area, established in 2009 according to Decree of the Minister of Marine Affairs and Fisheries Number KEP.67/MEN/2009, is managed under the Ministry of Marine Affairs and Fisheries (*Kementrian Kelautan dan Perikanan*), and locally by the National Marine Protected Areas Agency (*Balai Konservasi Kawasan Perairan Nasional*) Kupang. However, the purpose of the area is to motivate and integrate stakeholder participation and management in day-to-day use and conservation. Despite the common protected area, each island has its own distinct development issues and local leaders (all though cooperation and influence cross-over in some cases), and cooperation among local leaders, NGOs, businesses and government has not always been transparent or smooth. What the three islands have in common is their physical disconnection from the mainland, and local government oversight from the North Lombok Regency who is directly in charge of overall government issues. However, the national government is also involved directly in infrastructure development on the island. Arguably the islands are viewed as oases of revenue and employment, contrasted to the mainland areas in the regency which can be largely characterized as rural and agricultural, with low development standards. Providing government services on offshore islands is difficult, but is perhaps also not viewed as a priority to local officials who have seen near constant tourism and revenue growth on the islands with minimal inputs, where public funds may be better directed to much needed mainland economic and infrastructural needs. As the island is a very large source of revenue and economic prosperity in an otherwise under-developed region, corruption remains an issue that is regularly mentioned and likely exists across a variety of issues from land allocation and business permitting, tax revenue use as well as political and economic leverage by officials and influential business owners for personal gains. Navigating and improving governance processes in this context is necessary in many parts of the world, on an issue that is often unseen but influential in the processes of transitioning towards more sustainable systems.

On Gili Trawangan, change is now inevitable, but uncertain in the direction it will lead. Many of the actors who stay are likely to have an experience-based and mature sense of what makes the island's interrelated communities more resilient. As the pandemic unfolds, governance will evolve, and the community goals, relationships and networks will as well. The question of 'who governs' can be paired

This article is protected by copyright. All rights reserved.

Accepted Article with the question of 'what governs the island?'. Development on Gili Trawangan is immanent, it appears to have evolved from the aggregation of everyone's collective activities, rather than being interventionist and externally driven, for example, by state programs (Morse, 2008). Place attachment, knowledge and learning processes play strong roles. They exemplify how governance and resilience are embedded features of social systems, constantly evolving and adapting to what is happening. Networks and community cohesion are the fabric of social activities and beliefs, where leadership can help steer them towards taking more sustainable actions. However, leadership is also shared and iterative in relation to who has influence over who, an active political ecology with historical power dynamics. In conclusion, we believe this analysis has provided a baseline assessment of community resilience, and highlighted some of the main factors influencing the islands ability to cope with and emerge from the pandemic.

**Author affiliations**

**Stefan Partelow** Leibniz Centre for Tropical Marine Research (ZMT), Bremen, Germany.

**Marie Fujitani** Leibniz Centre for Tropical Marine Research (ZMT), Bremen, Germany and University of Bremen, Germany.

**Sian Williams** Gili EcoTrust, Gili Trawangan, Indonesia.

**Delphine Robbe** Gili EcoTrust, Gili Trawangan, Indonesia.

**Raditya Andrean Saputra** Indonesia Biru Foundation, Lombok, Indonesia.

**Corresponding Author**

**Stefan Partelow** Leibniz Centre for Tropical Marine Research (ZMT), Bremen, Germany. Email: stefan.partelow@leibniz-zmt.de

ORCID: https://orcid.org/0000-0002-7751-4005

**Data Availability Statement**

The data that support the findings of this study are available on request from the corresponding author. The data are not publicly available due to privacy or ethical restrictions.

**Acknowledgements**

We would like to thank the interviewees and the community of Gili Trawangan for their time and support in helping conduct this research during challenging times. SP and MF are grateful to the Leibniz Centre for Tropical Marine Research (ZMT) for funding support. MF acknowledges funding from the German Federal Ministry for Education and Science (BMBF) within the Transdisciplinary Science for Sustainable Tourism (TransTourism) project, funded through FONA (Research for Sustainable Development) program.
